# A look back at the first wave of COVID-19 in China: A systematic review and meta-analysis of mortality and health care resource use among severe or critical patients

**DOI:** 10.1371/journal.pone.0265117

**Published:** 2022-03-11

**Authors:** Mengmeng Zhang, Peng Hu, Xiaowei Xu, Jingwen Ai, Yang Li, Yun Bao, Wimonchat Tangamornsuksan, Alain Chan, Shelley Xie, Hao Hu, Shuting Liang, Wenhong Zhang, Feng Xie

**Affiliations:** 1 Department of Health Research Methods, Evidence, and Impact, McMaster University, Hamilton, Ontario, Canada; 2 Department of Infectious Disease, The Second Hospital Affiliated to Chongqing Medical University, Chongqing, China; 3 Department of Infectious Disease, The First Hospital Affiliated to Zhejiang University School of Medicine, Hangzhou, Zhejiang, China; 4 Department of Infectious Disease, Huashan Hospital, Fudan University, Shanghai, China; 5 Institute of Clinical Research and Evidence-Based Medicine, Gansu Provincial Hospital, Lanzhou, Gansu, China; 6 Princess Srisavangavadhana College of Medicine, Chulabhorn Royal Academy, Bangkok, Thailand; 7 China Medical Affairs, Gilead Sciences; 8 Global Value and Access, Gilead Sciences; 9 Global Medical Affairs Research, Gilead Sciences; 10 Centre for Health Economics and Policy Analysis, McMaster University, Hamilton, Ontario, Canada; Affiliated Hospital of Nantong University, CHINA

## Abstract

**Background:**

To investigate the mortality and health care resource use among patients with severe or critical coronavirus disease of 2019 (COVID-19) in the first wave of pandemic in China.

**Methods:**

We performed a systematic review and meta-analysis to investigate the mortality, discharge rate, length of hospital stay, and use of invasive ventilation in severe or critical COVID-19 cases in China. We searched electronic databases for studies from China with no restrictions on language or interventions patients received. We screened records, extracted data and assessed the quality of included studies in duplicate. We performed the meta-analysis using random-effect models through a Bayesian framework. Subgroup analyses were conducted to examine studies by disease severity, study location and patient enrolment start date. We also performed sensitivity analysis using various priors, and assessed between-study heterogeneity and publication bias for the primary outcomes.

**Results:**

Out of 6,205 titles and abstracts screened, 500 were reviewed in full text. A total of 42 studies were included in the review, of which 95% were observational studies (n = 40). The pooled 28-day and 14-day mortalities among severe or critical patients were 20.48% (7,136 patients, 95% credible interval (CrI), 13.11 to 30.70) and 10.83% (95% CrI, 6.78 to 16.75), respectively. The mortality declined over time and was higher in patients with critical disease than severe cases (1,235 patients, 45.73%, 95% CrI, 22.79 to 73.52 vs. 3,969 patients, 14.90%, 95% CrI, 4.70 to 39.57) and patients in Hubei compared to those outside Hubei (6,719 patients, 26.62%, 95% CrI, 13.11 to 30.70 vs. 244 patients, 5.88%, 95% CrI 2.03 to 14.11). The length of hospital stay was estimated at 18.48 days (6,847 patients, 95% CrI, 17.59 to 21.21), the 28-day discharge rate was 50.48% (3,645 patients, 95% CrI, 26.47 to 79.53), and the use of invasive ventilation rate was 13.46% (4,108 patients, 95% CrI, 7.61 to 22.31).

**Conclusions:**

Our systematic review and meta-analysis found high mortality among severe and critical COVID-19 cases. Severe or critical COVID-19 cases consumed a large amount of hospital resources during the outbreak.

## Background

Since the coronavirus disease 2019 (COVID-19) outbreak in December 2019 in Wuhan, China, there have been almost 300 million confirmed cases, with nearly 5.5 million lives lost worldwide [1]. It has been present in 192 countries or regions and crippled health care systems in many places [2–4].

Previous studies have shown that the infection spectrum of COVID-19, caused by the severe acute respiratory syndrome coronavirus 2 (SARS-CoV-2), ranges from asymptomatic to critical [5–7]. Among symptomatic patients, 80% developed mild or moderate disease, while approximately 15% had severe disease and 5% critical disease [5, 7]. Compared to mild or moderate cases, severe or critical ones tended to have much higher mortality. In February 2020, China Center for Disease Control and Prevention (CCDC) reported that the case fatality rate (CFR) among critical cases was as high as 49.0%, while there was no death among mild or moderate cases [8]. The mortality rate varied widely across studies and countries. Studies from China reported that the mortality of critical COVID-19 cases ranged from 16% to 78% [9–11]. A study from Italy reported a mortality rate of 26% among those admitted to ICU, while a US study reported 67% for critical cases [12, 13].

Patients with severe or critical illness may require advanced medical services such as oxygen/ventilatory support, intensive care unit (ICU) admission, and the use of extracorporeal membrane oxygenation (ECMO) [6, 7]. During the pandemic, health care resources needed for caring severe or critical cases were high and imposed an enormous pressure on health care systems worldwide [2, 14]. The use of intensive care resources also varied across studies [15–19]. The percentage of severe cases on invasive ventilation ranged from 5.6% to 51.7% [15–18].

Large between-study variations increase the difficulty of understanding the true impact of COVID-19 on mortality and resource use. Previously published reviews have been primarily focused on all COVID-19 patients combined [20–24]. However, it is important and necessary to understand the impact of COVID-19 among severely or critically ill patients. During the initial outbreak in China, many patients were infected with severe or critical diseases of COVID-19 and a large amount of COVID-19 research was published with enormous heterogeneity in methods and results. After the first wave, the spread of the virus had been well under control in China. Therefore, a thorough review of the totality of the evidence collected during this period is needed to better assess the impact of COVID-19 on severe or critical patients and the health care systems.

## Methods

### Search strategy and selection criteria

We performed a systematic literature search in multiple electronic databases for studies published from January 1 to October 2, 2020. We searched OVID MEDLINE® and Embase and a few major Chinese databases: China National Knowledge Infrastructure (CNKI), Wanfang Data, and Chinese Medical Association Publishing House (CMAPH). Since our objective was to synthesize outcomes reported in studies among severely or critically ill adult COVID-19 patients in China, we set no limit on treatments patients received; and only used the disease terms for COVID-19, including coronavirus, SARS-CoV-2 and COVID-19, and study geography terms for China. Non-human studies and case reports were excluded from the search. The search strategies are presented in S1 Appendix.

We included randomized controlled trials (RCTs), cohort studies, case-control studies and case series studies that reported any of the four primary outcomes, including mortality, the discharge rate, the length of hospital stay (LOS), and the use of invasive ventilation among patients with confirmed severe or critical COVID-19. Studies focusing on coronavirus diseases other than COVID-19, patients with suspected but unconfirmed COVID-19, and pregnant women or children were excluded. Studies on mixed populations where outcomes of COVID-19 for severe or critical patients were not reported separately were excluded. Studies that did not report any information about the study duration or the follow-up time for mortality or discharge were also excluded. When multiple papers were published based on the studies conducted in the same hospitals during the outbreak, we excluded studies that did not report sufficient study site details (e.g., province, city, and name of hospitals), the data collection timeframe or the patient enrolment timeframe to avoid double counting. Studies with less than 50 severe/critical patients were also excluded. There was no language limit in the search.

### Review process and data extraction

The title and abstract screening, the full-text review, and the risk of bias assessment were all conducted by two reviewers independently and in duplicate. Data were extracted by the first reviewer using a pre-designed extraction form and examined by the second reviewer. We assessed the risk of bias of included cohort studies, case-control studies, and randomized controlled trials (RCTs) using the modified tools developed by Busse and Guyatt [25, 26]. Any difference was resolved through group discussions until a consensus was reached. The study characteristics, demographic and clinical characteristics of patients, and the primary and secondary outcomes were extracted.

### Outcomes

The primary outcomes of interest were mortality, LOS, the discharge rate, and the rate of using invasive ventilation. In this review, the mortality was referred to as the case fatality ratio (CFR), estimated as the proportion of confirmed cases who died from COVID-19 [27]. The discharge rate was defined as the proportion of hospitalized patients discharged alive. LOS referred to the number of days that patients stayed in the hospital, from their admissions to death, discharge, or end of follow-up, whichever came first. Invasive ventilation rate was defined as the proportion of enrolled patients who had used invasive ventilation during their hospitalization. The secondary outcomes included the use of non-invasive ventilation and ECMO rates.

### Data analysis

We conducted the meta-analysis using a Bayesian framework based on the technical guidance from the National Institute for Health and Care Excellence Decision Support Unit (NICE DSU) [28]. This modeling framework can account for possible between-study variation, which is an essential consideration in our meta-analysis of studies covering various time frames, locations, and phases of the pandemic in China [29]. We used the cloglog link model to analyze the mortality and the discharge rate, the identity link model for LOS, and the logit link model for the invasive ventilation rate, the use of non-invasive ventilation rate and the use of ECMO rate [30].

There were a few assumptions in our analysis. For the mortality and the discharge rate, the follow-up timepoints might not be reported in all studies. Therefore, the median or mean LOS, if available, was used as a proxy for the follow-up time. In the cloglog link model synthesizing the 28-day and 14-day CFRs and discharge rates the median follow-up time was included as a variable. A few published studies found that the longer the follow-up time, the larger the number of events, which supported the assumption in the cloglog link model [31, 32].

In the meta-analysis, we pooled the data by combining eligible patients from separate groups in comparative studies or single-arm studies. When multiple studies reported the same outcome from patients of the same disease severity from the same hospital, only the study with the largest population size was included in the analysis. We assessed heterogeneity using the *I*^*2*^ statistic [29]. We performed subgroup analyses by disease severity (i.e., severe vs critical), enrollment start date (Dec 2019, Jan 2020, and Feb 2020), and locations (i.e., Hubei, where Wuhan is the capital city vs other provinces) in China. We conducted the Egger’s test to assess the publication bias for meta-analyses of the primary outcomes [33]. We performed a sensitivity analysis to examine the impact of various uniform and inverse-gamma priors for the variance of the estimates for the primary outcomes.

We conducted all the Bayesian meta-analyses in R (version 4.0.5) using the R2jags package and the Just Another Gibbs Sampler (JAGS) program. Three chains were run using the Markov chain Monte Carlo (MCMC) algorithm, each with 50,000 iterations and a burn-in of 25,000 iterations (and a thinning of 10). The convergence of the chains was determined by examining the trace and density plots of the parameters. All results are presented using medians with 95% credibility interval (CrI).

## Results

**Fig 1:**
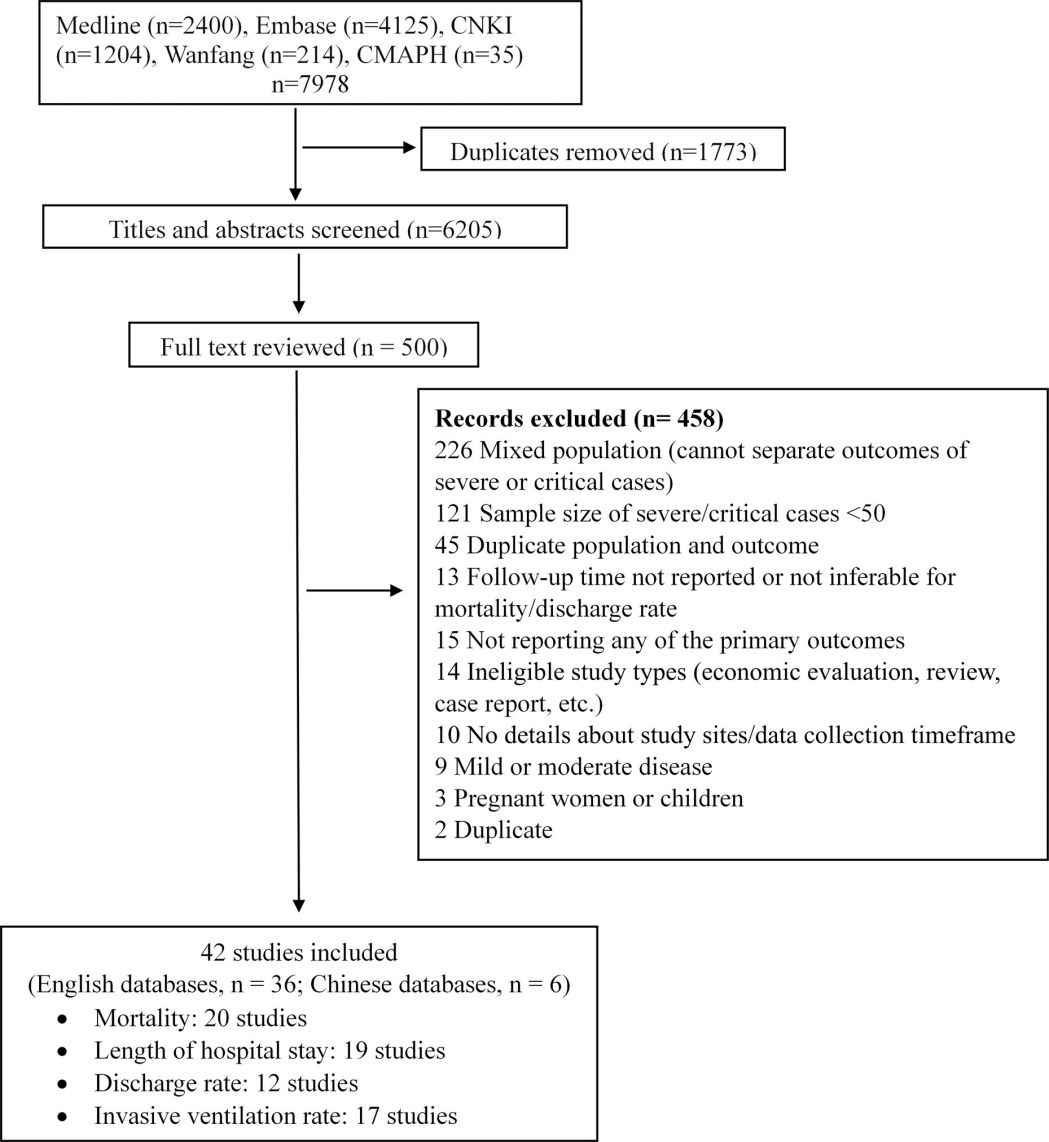
Flow diagram of literature screening and selection.

Fig 1 shows the details of the study selection process. A total of 7,978 records were identified from the literature search. After removing the duplicates, 6,205 titles and abstracts were screened. Of the 500 papers reviewed in full text, 42 were included for our systematic review and meta-analysis, with 36 from English databases and six from Chinese databases [15–19, 31, 34–69]. Forty (95%) studies were observational studies, 25 (60%) single-center, and 35 (83%) enrolled patients in Hubei Province. All included studies enrolled patients from January 2020 to April 2020 which was the first wave of the pandemic in China before Wuhan lifted the lockdown [70]. Thirty-six studies (85%) started the patient enrolment between January and February 2020. Eighteen (43%) studies included both severe and critical cases, 16 (38%) severe cases only, and eight (19%) critical cases only (Table 1). Among the 18 studies that included both severe and critical cases, the proportion of severe cases ranged from 36% to 92.6%. The number of severe or critical cases ranged from 50 to 1,763. Thirty-three (79%) studies assessed disease severity following the Chinese guidelines for diagnosis and treatment of COVID-19 released by the National Health Commission. The mean age of patients ranged from 52.5 to 70.7 years. The proportion of males ranged from 44.9% to 68.5%. The numbers of studies included in the meta-analysis were 20 for CFR, 19 for LOS, 12 for the discharge rate and 17 for the rate of using invasive ventilation (Fig 1 & S2 Appendix). Thirty-seven studies reported the comorbidities of patients (S3 Appendix). The risk of bias of included studies was low (S4 Appendix). The results for heterogeneity and the publication bias analyses are presented in S5 Appendix.

**Table 1:** Characteristics of studies included in the meta-analysis in patients with severe or critical COVID-19 (n = 42).

Study name	Number of patients	Design	Location	Enrolment timeframe	Definitions for severe/critical cases	Severity of disease	Severe, n(%)	Male, n(%)	Age, mean ± SD
*Guan et al. 2020 (#5611) [18]	173	Retrospective cohort study	China, 30 provinces	Dec 11, 2019 to Jan 29, 2020	American Thoracic Society guidelines for community-acquired pneumonia	Severe	173 (100)	100 (57.8)	52.5 ± 18.7
Tang et al. 2020 (#10371) [34]	73	Case-control study	Hubei, Wuhan	Dec 24, 2019 to Feb 7, 2020	NR	Critical	0	45 (61.6)	63.6 ± 11.7
Xu et al. 2020 (#9329) [35]	107	Retrospective cohort study	Hubei, Wuhan	Dec 26, 2019 to Mar 1, 2020	Chinese guideline for the diagnosis and treatment of COVID-19 †	Mixed	45 (42.1)	73 (68.2)	62.7 ± 13.9
Liu et al. 2020 (#3961) [36]	349	Retrospective cohort study	Hubei, Wuhan	Dec 29, 2019 to Feb 28, 2020	Chinese guideline for the diagnosis and treatment of COVID-19 (6^th^ edition)	Severe	349 (100)	206 (59)	61.1 ± 15
*Wu et al. 2020 (#2644) [37]	1763	Retrospective cohort study	Hubei, Wuhan	Dec 26, 2019 to Mar 15, 2020	Severe cases: use of oxygen therapy during hospital stay	Mixed	1514 (85.9)	871 (49.4)	60.6 ± 14.7
*Xu et al. 2020 (#4838) [38]	239	Retrospective cohort study	Hubei, Wuhan	Jan 12, 2020 to Feb 3, 2020	Critical cases: admission to ICU, use of mechanical ventilation, or FiO2 ≥ 60%	Critical	0	143 (59.8)	62.5 ± 13.3
Zhang et al. 2020 (#10522) [39]	107	Retrospective cohort study	Hubei, Wuhan	Jan 1, 2020 to Feb 1, 2020	Chinese guideline for the diagnosis and treatment of COVID-19 (6^th^ edition)	Mixed	NR	62 (57.9)	59.8 ± 13
Liu et al. 2020 (#3799) [40]	79	Retrospective cohort study	Hubei, Wuhan	Jan 22, 2020 to Mar 6, 2020	Chinese guideline for the diagnosis and treatment of COVID-19 (7^th^ edition)	Mixed	62 (78.5)	50 (63.3)	62.5 ± 12.6
Cai et al. 2020 (#4791) [15]	58	Retrospective cohort study	Guangdong, Shenzhen	Jan 11, 2020 to Feb 6, 2020	Chinese guideline for the diagnosis and treatment of COVID-19 (5^th^ edition)	Severe	58 (100)	39 (67.2)	60.9 ± 7.8
*Ma et al. 2020 (#2629) [43]	82	Retrospective cohort study	Hunan, China	Jan 23, 2020 to Mar 8, 2020	Chinese guideline for the diagnosis and treatment of COVID-19 (6^th^ edition)	Severe	82 (100)	48 (58.5)	56.9 ± 15.5
*Zhang et al. 2020 (#4274) [64]	539	Retrospective cohort study	Hubei, Wuhan	Jan 12, 2020, to Feb 7, 2020	Chinese guideline for the diagnosis and treatment of COVID-19 (7^th^ edition)	Severe	539 (100)	NR	NR
Wang et al. 2020 (#10019) [63]	239	Retrospective cohort study	Hubei, Wuhan	Jan 1, 2020 to Feb 6, 2020	Chinese guideline for the diagnosis and treatment of COVID-19 (6^th^ edition)	Mixed	159 (66.5)	NR	NR
Xu et al. 2020 (#3248) [47]	50	Retrospective cohort study	Sichuan, Hubei	Jan 17, 2020 to Mar 2, 2020	Chinese guideline for the diagnosis and treatment of COVID-19 (7^th^ edition)	Mixed	18 (36)	31 (62)	59.7 ± 14.7
Liu et al. 2020 (#2726) [42]	957	Retrospective cohort study	Hubei, Wuhan	Jan 27, 2020 to Mar 21, 2020	Chinese guideline for the diagnosis and treatment of COVID-19 (7^th^ edition)	Mixed	689 (72)	525 (54.9)	64.2 ± 12
*Chen et al. 2020 (#4319) [53]	51	Retrospective cohort study	Hebei, 13 hospitals	Jan 22, 2020 to Mar 25, 2020	Chinese guideline for the diagnosis and treatment of COVID-19 (7^th^ edition)	Mixed	31 (60.8)	27 (52.9)	59 ± 13.7
Yu et al. 2020 (#10679) [65]	53	Retrospective cohort study	Tianjin	Jan 21, 2020 to Mar 15, 2020	Chinese guideline for the diagnosis and treatment of COVID-19 (7^th^ edition)	Mixed	NR	32 (60.4)	57.1 ± 14.4
*Wang et al. 2020 (#3550) [31]	236	RCT	Hubei, Wuhan	Feb 6, 2020 to Mar 12, 2020	NR	Severe	236 (100)	140 (59.3)	63.3 ± 12
*Zhu et al. 2020 (#8898) [17]	102	Retrospective cohort study	Hubei	Feb to Mar 2020	Chinese guideline for the diagnosis and treatment of COVID-19 (7^th^ edition)	Severe	102 (100)	59 (57.8)	69.6 ± 14
Xia et al. 2020 (#8941) [62]	1568	Retrospective cohort study	Hubei, Wuhan	Feb 4, 2020 to Mar 30, 2020	Chinese guideline for the diagnosis and treatment of COVID-19 (6^th^ edition)	Mixed	1420 (90.6)	797 (50.8)	60.9 ± 13.3
Liu et al. 2020 (#4147) [50]	311	Retrospective cohort study	Hubei, Wuhan	Feb 8, 2020 to Apr 15, 2020	NR	Mixed	288 (92.6)	NR	NR
Zhang et al. 2020 (#3150) [48]	75	Retrospective cohort study	Hubei, Wuhan	Jan 24, 2020 to Mar 26, 2020	Chinese guideline for the diagnosis and treatment of COVID-19 (7^th^ edition)	Critical	0	59 (78.7)	64.6 ± 9.4
Pan et al. 2020 (#8173) [59]	124	Case-control study	Hubei, Wuhan	Jan 27, 2020 to Mar 19, 2020	Chinese guideline for the diagnosis and treatment of COVID-19 (6^th^ edition)	Severe	124 (100)	85 (68.5)	67.9 ± 10.9
Yu et al. 2020 (#2842) [44]	864	Retrospective cohort study	Hubei, Wuhan	Jan 14, 2020 to Feb 28, 2020	Chinese guideline for the diagnosis and treatment of COVID-19 (5^th^ - 6^th^ edition)	Severe	864 (100)	454 (52.5)	64.1 ± 12
Xiong et al. 2020 (#11162) [66]	305	Retrospective cohort study	Hubei, Wuhan	Jan 2, 2020 to Feb 15, 2020	Chinese guideline for the diagnosis and treatment of COVID-19 (5^th^ edition)	Mixed	166 (54.4)	171 (56.1)	61.9 ± 13.4
Yang et al. 2020 (#5850) [57]	52	Retrospective cohort study	Hubei, Wuhan	Dec 24, 2019, to Jan 26, 2020	WHO interim guideline	Critical	0	35 (67.3)	59.7 ± 13.3
*Zhou et al. 2020 (#4245) [55]	195	Case series	Hubei	Jan 5, 2020 to Apr 3, 2020	WHO interim guideline	Critical	0	130 (66.7)	65.1 ± 15.5
Cheng et al. 2020 (#11809) [67]	181	Retrospective cohort study	Hubei, Wuhan	Jan 1, 2020 to Feb 6, 2020	Chinese guideline for the diagnosis and treatment of COVID-19 (5^th^ edition)	Severe	181 (100)	99 (54.7)	55.4 ± 14.3
Shao et al. 2020 (#6230) [19]	136	Retrospective cohort study	Hubei, Wuhan	Jan 15, 2020 to Feb 25, 2020	NR	Severe	136 (100)	90 (66.2)	69 ± 11.9
*Xie et al. 2020 (#4193) [52]	733	Retrospective cohort study	Hubei, Guangdong, and Jiangsu	January 1, 2020 to February 29, 2020	Chinese guideline for the diagnosis and treatment of COVID-19 (7^th^ edition)	Critical	0	477 (65.1)	63.4 ± 13
*Ma et al. 2020 (#8986) [60]	72	Retrospective cohort study	Chongqing	Jan 2020 to Mar 2020	Chinese guideline for the diagnosis and treatment of COVID-19 (7^th^ edition)	Mixed	46 (63.9)	40 (55.6)	60.7 ± 13.8
Wang et al. 2020 (#8872) [61]	59	Retrospective cohort study	Hubei, Wuhan	Feb 9, 2020 to Mar 5, 2020	Chinese guideline for the diagnosis and treatment of COVID-19 †	Severe	59 (100)	28 (47.5)	53 ± 11.7
*Huang et al. 2020 (#2956) [45]	60	Retrospective cohort study	Jiangsu	Jan 24, 2020 to Apr 20, 2020	Chinese guideline for the diagnosis and treatment of COVID-19 (5^th^ edition)	Severe	60 (100)	35 (58.3)	57.1 ± 15.8
Zhong et al. 2020 (#10923) [68]	583	Retrospective cohort study	Hubei, Wuhan	Jan 1, 2020 to Feb 20, 2020	Chinese guideline for the diagnosis and treatment of COVID-19 (7^th^ edition)	Mixed	445 (76.3)	293 (50.3)	61.8 ± 14.2
Yang et al. 2020 (#10707) [69]	301	Retrospective cohort study	Hubei, Wuhan	Jan 12, 2020 to Mar 7, 2020	Chinese guideline for the diagnosis and treatment of COVID-19 (7^th^ edition)	Mixed	254 (84.4)	135 (44.9)	63.1 ± 13.6
Chen et al. 2020 (#3846) [16]	681	Retrospective cohort study	Hubei, Wuhan	Jan 3, 2020 to Apr 9, 2020	Chinese guideline for the diagnosis and treatment of COVID-19	Severe	681 (100)	362 (53.2)	62.6 ± 12.3
*Xu et al. 2020 (#3634) [49]	198	Retrospective cohort study	Shanghai, Wuhan and Anhui	January 1, 2020 to March 8, 2020	Chinese guideline for the diagnosis and treatment of COVID-19 (5^th^ edition)	Mixed	85 (42.9)	128 (64.6)	61.8 ± 11.9
*Zhang et al. 2020 (#4326) [54]	78	Retrospective cohort study	Zhejiang	Jan 17, 2020 to Feb 12, 2020	Chinese guideline for the diagnosis and treatment of COVID-19 (6^th^ edition)	Mixed	61 (78.2)	52 (66.7)	55.5 ± 12.5
Zhang et al. 2020 (#4544) [56]	136	Retrospective cohort study	Hubei, Wuhan	Jan 28, 2020 to Feb 21, 2020	Chinese guideline for the diagnosis and treatment of COVID-19 (6^th^ edition)	Critical	0	86 (63.2)	67.4 ± 13.3
*Tian et al. 2020 (#3869) [51]	148	Retrospective cohort study	Hubei, Wuhan	Jan 13, 2020 to Mar 18, 2020	WHO interim guideline and Chinese guideline for the diagnosis and treatment of COVID-19 (7^th^ edition)	Severe	148 (100)	81 (54.7)	62.7 ± 8.8
Li et al. 2020 (#3139) [46]	173	Case series	Hubei, Wuhan	Jan 15, 2020 to Mar 15, 2020	WHO interim guideline and Chinese guideline for the diagnosis and treatment of COVID-19 (5^th^ edition)	Severe	173 (100)	97 (56.1)	69.5 ± 10.6
*Li et al. 2020 (#2564) [41]	103	RCT	Hubei, Wuhan	Feb 14, 2020 to April 1, 2020	Chinese guideline for the diagnosis and treatment of COVID-19†	Mixed	45 (43.7)	60 (58.3)	70.7 ± 12.1
*Yu et al. 2020 (#6374) [58]	226	Cross-sectional study	Hubei, Wuhan	Feb 26, 2020 to Feb 27, 2020	Chinese guideline for the diagnosis and treatment of COVID-19 (5^th^ edition)	Critical	0	139 (61.5)	62.6 ± 10.3

Abbreviations: COVID-19: coronavirus disease; SD: standard deviation; RCT: randomized controlled trial; NR: not reported.

* indicates the study involves patients from multiple centers; otherwise, the study involves a single center.

† indicates that the study did not specify the guidelines they used for the diagnosis of severe/critical cases, but the definitions they used were consistent with the mentioned guideline.

### Primary outcomes

#### 28-day and 14-day mortalities

**Fig 2:**
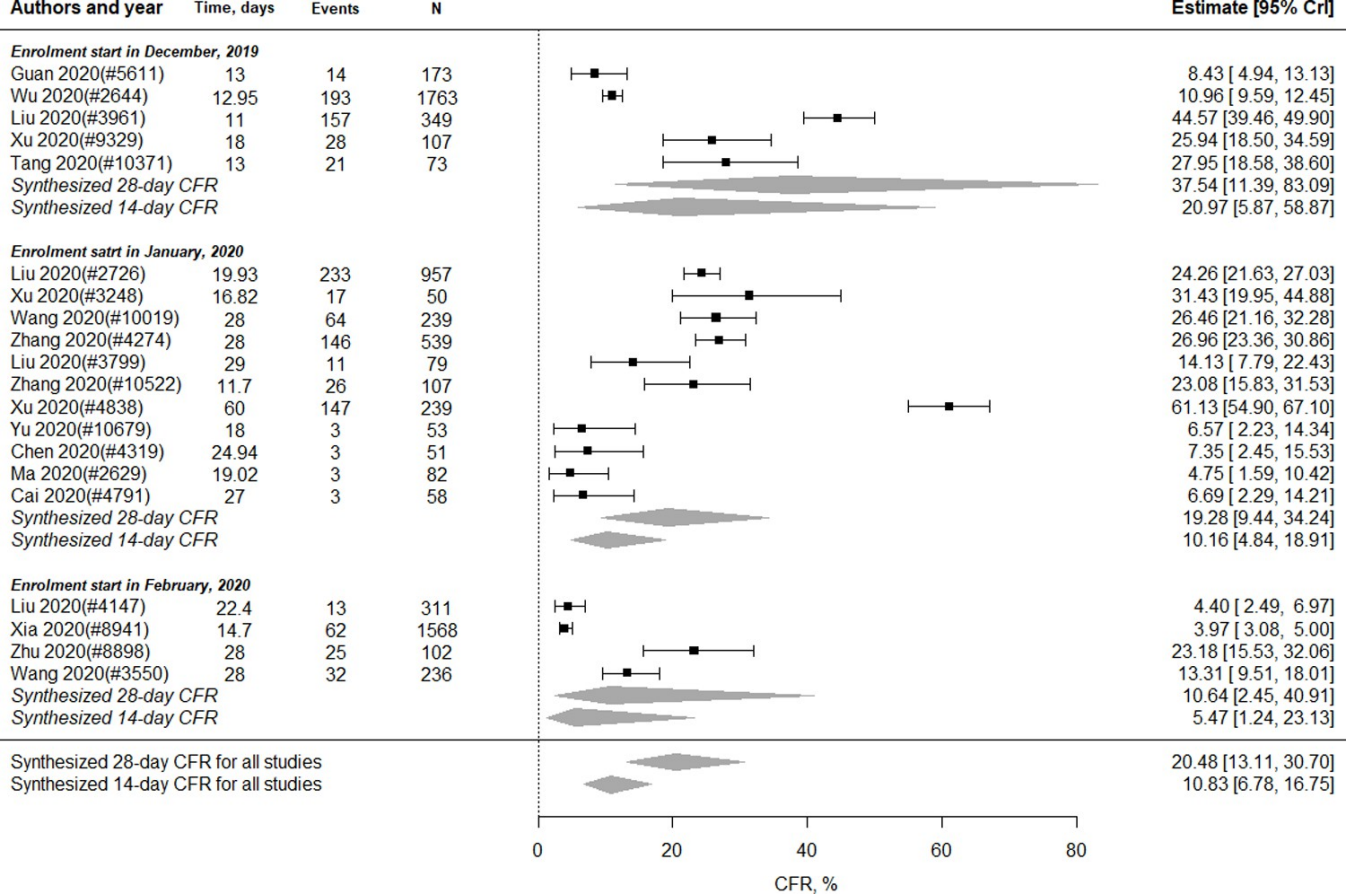
28-day and 14-day CFRs for severe or critical COVID-19 patients in China.

There were 20 studies (n = 7,136 patients) included in the meta-analysis of CFR. As shown in Fig 2, the pooled 28-day CFR was 20.48% (95% CrI 13.11 to 30.70) and 14-day CFR 10.83% (95% CrI 6.78 to 16.75). Nine studies (n = 3,969 patients) and four studies (n = 1,235 patients) reported the CFR of severe and critical cases, respectively. Five (n = 2,531 patients), 11 (n = 4,361 patients) and four studies (n = 244 patients) reported the CFR for studies starting their patient enrolment in December 2019, January 2020, and February 2020, respectively. As shown in Fig 2, for studies starting patient enrolment in December, the pooled 28-day CFR was 37.54% (95% CrI 11.39 to 83.09) and the 14-day CFR was 20.97% (95% CrI 5.87 to 58.87); for studies starting patient enrolment in January, the pooled 28-day CFR was 19.28% (95% CrI 9.44 to 34.24) and the 14-day CFR was 10.16% (95% CrI 4.84 to 18.91); and for studies starting the patient enrolment in February, the pooled 28-day CFR was 10.64% (95% CrI 2.45 to 40.91) and the 14-day CFR 5.47% (95% CrI 1.24 to 23.13).

Figs A and B in S6 Appendix show the analysis results by severity and location. In Fig A in S6 Appendix, the pooled 28-day CFR of severe cases was 14.90% (95% CrI, 4.70 to 39.57), and the 14-day CFR was 7.75% (95% CrI, 2.38 to 22.26). For critical cases, the pooled 28-day CFR was 45.73% (95% CrI, 22.79 to 73.52), and 14-day CFR was 26.33% (95%CrI 12.13 to 48.54). Fifteen studies (n = 6,719 patients) and four studies (n = 244 patients) reported the CFR in patients in and outside Hubei, respectively. As shown in Fig B in S6 Appendix, the pooled 28-day CFR in patients in Hubei was 26.62% (95%, 16.93 to 40.05), and the 14-day CFR 14.34% (95% CrI 8.86 to 22.57). In contrast, the pooled 28-day CFR in patients outside Hubei was 5.88% (95% CrI, 2.03 to 14.11), and the 14-day CFR was 2.98% (95% CrI, 1.02 to 7.32).

#### Length of hospital stay (LOS)

**Fig 3:**
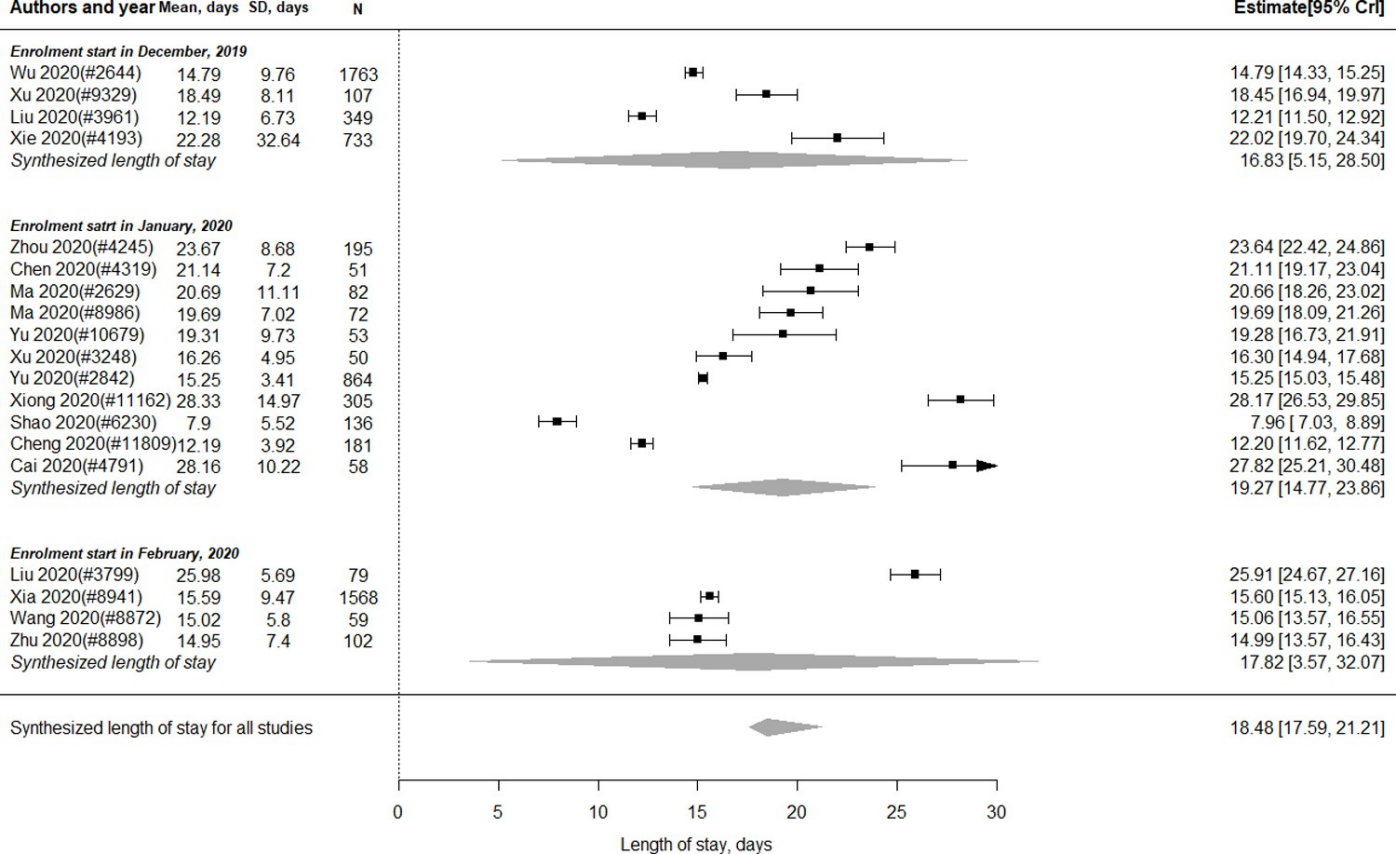
Length of hospital stay for severe or critical COVID-19 patients in China.

There were 19 studies (n = 6,807 patients) included in the meta-analysis of LOS. As shown in Fig 3, the estimated LOS was 18.48 days (95% CrI 17.59 to 21.21). Among those reported LOS, four (n = 2,952 patients), 11 (n = 2,047 patients) and four studies (n = 1,808 patients) started their patient enrolment in December 2019, January 2020, and February 2020, respectively. As shown in Fig 3, the pooled LOS for studies starting patient enrolment in December, January and February were 16.83 days (95% CrI 5.15 to 28.50), 19.27 days (95% CrI 14.77 to 23.86) and 17.82 days (95% CrI 3.57 to 32.07), respectively.

Ten studies (n = 3,518 patients) and four studies (n = 1,250 patients) reported the LOS of severe and critical cases, respectively. As shown in Fig C in S6 Appendix, the pooled LOS of severe and critical cases was 16.56 days (95% CrI 11.84 to 21.38) and 18.76 days (95% CrI 5.72 to 32.35), respectively.

#### Discharge rate

**Fig 4:**
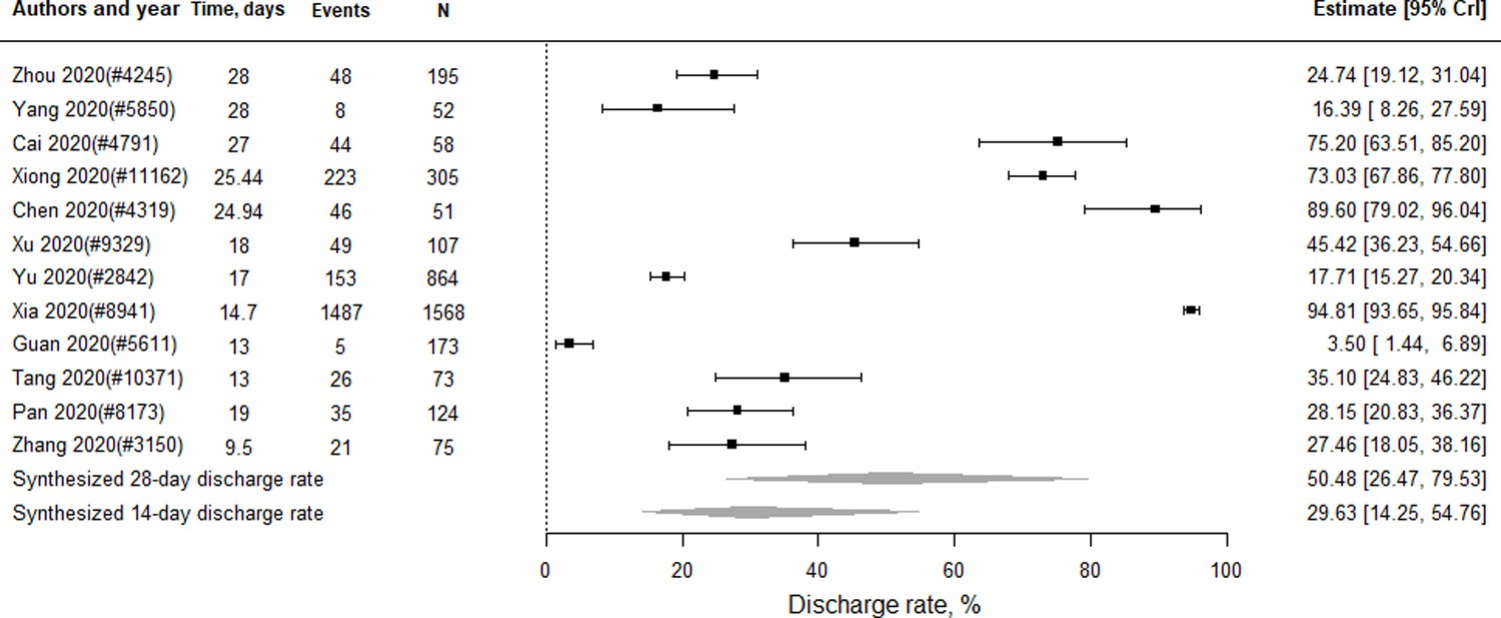
Discharge rate for severe or critical COVID-19 patients in China.

There were 12 studies (n = 3,645 patients) included in the meta-analysis of discharge rate. As shown in Fig 4, the pooled 14-day discharge rate was 29.63% (95% CrI 14.25 to 54.76), and the 28-day discharge rate was 50.48% (95% CrI 26.47 to 79.53). Four (n = 1,219 patients) and five studies (n = 453 patients) reported the discharge rate of severe and critical cases, respectively. As shown in Fig D in S6 Appendix, the pooled 14-day and 28-day discharge rates were 16.12% (95% CrI 1.51 to 83.22) and 29.64% (95% CrI 3.01 to 97.18), respectively, for severe cases. The pooled 14-day and 28-day discharge rates for critical cases were 16.65% (95% CrI 4.10 to 51.75) and 30.53% (95% CrI 8.04 to 76.72).

#### Invasive ventilation rate

**Fig 5:**
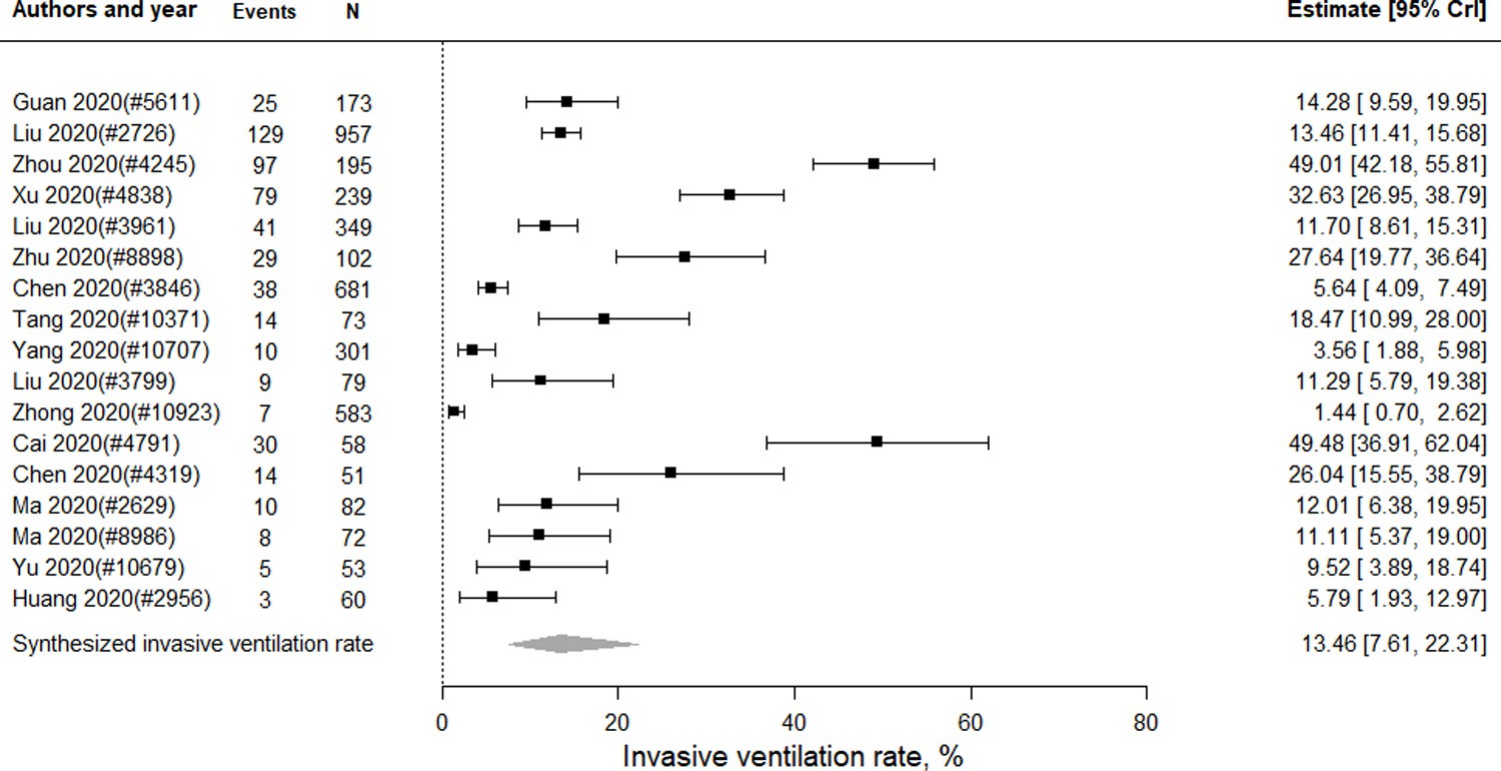
The use of invasive ventilation rate by severe or critical COVID-19 patients in China.

Seventeen studies (n = 4,108 patients) were included in the meta-analysis of the rate of using invasive ventilation. As shown in Fig 5, the pooled invasive ventilation rate was 13.46% (95% CrI 7.61 to 22.31). Eight (n = 1,653 patients) and three studies (n = 1,154 patients) reported the invasive ventilation rate of severe and critical cases, respectively. As shown in Fig E in S6 Appendix, the pooled use of invasive ventilation rate was 14.42% (95% CrI 6.27 to 29.65) for severe cases and 47.86% (95% CrI 20.33 to 77.56) for critical cases.

#### Secondary outcomes

There were 15 studies (n = 3,894 patients) included in the meta-analysis of the use of non-invasive ventilation rate, and the pooled rate was 25.38% (95% CrI 18.45 to 33.40). There were 12 studies (n = 2,430 patients) included in the meta-analysis of the use of ECMO rate, and the pooled rate was 2.31% (95% CrI 1.06 to 4.25).

### Sensitivity analysis

When using different priors for the population variance, the results of meta-analyses for primary outcomes were similar (S7 Appendix).

## Discussions

Our systematic review and meta-analysis revealed high mortality rates among severely and critically ill patients with COVID-19 in China, while the rate declined over time. Most studies were conducted in Hubei, China, and the mortality in Hubei was significantly higher than that in other provinces. The length of stay in hospital was estimated at 18 days, similar across the subgroups. The discharge rate was similar in severe and critical cases. Patients with critical disease required more intensive care resources than those with severe disease.

The International Severe Acute Respiratory and emerging Infections Consortium (ISARIC) COVID-19 report as of November 9, 2020, involved 95,966 confirmed cases with longer than 14 days of follow-up across 42 countries [71]. According to this report, most cases were from the UK; 20% of all cases were admitted to ICU or high dependency unit (HDU). The mortality rate among patients admitted to ICU/HCU was 35% [71]. On the other hand, the US Centers for Disease Control and Prevention (CDC) revealed a mortality of 65.2% among hospitalized patients as of April 14, 2021 [72]. In the report from China CDC involving 2,087 critical cases across China until February 11, 2020, the mortality was 49.0%, slightly higher than our pooled estimates of 45.73% among critical cases [8]. Consistent with the findings from a cohort study in the UK, our estimated mortality rates in severe and critical cases fell over time [73]. This improvement in survival over time could be attributed to a few different factors, including temporal changes in COVID-19 disease severity at admission, improved treatment experience of clinicians, and improved hospital capacity over time [73, 74].

A few studies have estimated the LOS of COVID-19 patients in China [75–77]. A systematic review estimated the median LOS of patients, including mild and moderate cases, was 14 days (IQR 10–19) in China [75]. It is lower than our estimated LOS in severe or critical patients, consistent with previous review findings that severe cases tend to have longer LOS than mild or moderate cases [75]. Our estimates of the 28-day discharge rate among severe and critical cases were both around 30%, which was lower than the estimate for the whole group of patients. One possible reason is that some studies included in the whole group meta-analysis of discharge rate had a mixed population where the discharge rates for severe and critical cases were not reported separately, and they had relatively large sample size and high discharge rate [62, 66]. For example, the study by Xia et al. enrolled 1,568 patients with a discharge rate of 95% [62]. Another possible reason is the heterogeneity between studies due to the various discharge criteria, availability of hospital beds and diverse patient characteristics in these studies [20, 78].

Our systematic review has a few strengths. First, it captured a large number of published studies with more complete data in China during the first wave of the pandemic. For the rest of the year, the virus has been well under control. Previous reviews were limited in that most of them were performed during the outbreak in China, with limited published or mature data included in their analyses [20–24]. With the outbreak ongoing, the number of discharged cases, hospitalized cases, and deaths changed over time. These reviews’ mortality rates might be biased because of the unknown and unpredictable prognosis among the large number of hospitalized patients [79]. Also, the inclusion of unpublished non-peer-reviewed reports in previous reviews could bias the estimates [22, 23]. Another strength is that we incorporated the follow-up time into our estimate of mortality using the cloglog link model, which allows us to include more studies with heterogeneous follow-up times in the analysis. Finally, our study’s subgroup analysis provides evidence about the variation of health outcomes and medical resource use across different phases of the pandemic and different locations in China.

A few limitations in our review are worth noting. A major limitation is that 95% of included studies are observational and retrospective. Second, we have found substantial heterogeneity between included studies with respect to their data collection timeframes, locations, and patient characteristics. Third, we only included published reports and most studies included in our studies were from Hubei. Patients outside Hubei might be underreported. Fourth, although we have used median or mean LOS as a proxy for the follow-up time, there is a risk of inaccuracy when estimating the follow-up time. Given only a small number of studies used proxies, the impact of this process on the pooled results was minimal.

The findings from our study could be useful for decisionmakings concerning disease management and resource allocation to design an effective preparedness plan for the ongoing and future pandemics [80]. Using the estimates of resource use among the severe and critical cases and an infection rate among populations, the required medical resources for a particular group of people could be estimated. We could have more effective preparedness and response for the ongoing and potential future pandemics.

## Conclusions

Our systematic review and meta-analysis found high mortality among severe or critical COVID-19 patients, while it declined over time. These patients consumed a large amount of hospital resources during the outbreak.

## Supporting information

S1 Appendix Search strategy and history. (DOCX)

S2 Appendix Number of studies and characteristics of patients included in the meta-analysis. (DOCX)

S3 Appendix Comorbidities of patients included in the meta-analysis. (DOCX)

S4 Appendix Quality assessment of included studies. Table A. Risk of bias of included cohort studies. Table B. Risk of bias of included case-control studies. Table C. Risk of bias of included randomized controlled trials.
(DOCX)

S5 Appendix Heterogeneity and publication bias assessment results. (DOCX)

S6 Appendix Subgroup analysis results. Fig A 28-day and 14-day CFRs among patients with severe vs critical COVID-19. Fig B 28-day and 14-day CFRs in Hubei vs other locations in China. Fig C Length of hospital stay among patients with severe vs critical COVID-19. Fig D Discharge rate among patients with severe vs critical COVID-19. Fig E The use of invasive ventilation rate among patients with severe vs critical COVID-19.
(DOCX)

S7 Appendix Sensitivity analyses with different priors. (DOCX)

S8 Appendix PRISMA checklist. (DOCX)
