# Supplementary material for: A look back at the first wave of COVID-19 in China: A systematic review and meta-analysis of mortality and health care resource use among severe or critical patients
Source: PLoS One. 2022 Mar 11;17(3):e0265117. doi: 10.1371/journal.pone.0265117 (PMC8916647; doi:10.1371/journal.pone.0265117)
Supplement: S1 Appendix — (DOCX) [file pone.0265117.s001.docx]

**S1 Appendix. Search strategy and history**

**Database: Ovid MEDLINE(R)**

**Search Strategy:**

--------------------------------------------------------------------------------

1 exp coronavirus/ or exp coronavirus infections/ (40160)

2 SARS-CoV-2.mp. (7663)

3 (coronavirus$ or corona virus$).mp. (38251)

4 (Covid-19 or Covid19).mp. (26880)

5 nCoV.mp. (745)

6 global health emergency.mp. (131)

7 or/1-6 (42374)

8 exp Animals/ not exp Humans/ (2562283)

9 (editorial or "case reports").pt. (1599385)

10 (case stud$ or case report$).ti. (163923)

11 or/8-10 (4151089)

12 7 not 11 (31413)

13 limit 12 to dt=20200101-20201231 (22726)

14 exp China/ or exp Chinese/ (230360)

15 13 and 14 (2412)

16 remove duplicates from 15 (2400)

**Database: Embase**

**Search Strategy:**

--------------------------------------------------------------------------------

1 exp coronavirus/ or exp coronavirus infections/ (29822)

2 SARS-CoV-2.mp. (16521)

3 (coronavirus$ or corona virus$).mp. (74885)

4 (Covid-19 or Covid19).mp. (49173)

5 nCoV.mp. (1247)

6 global health emergency.mp. (258)

7 or/1-6 (83131)

8 exp Animals/ not exp Humans/ (4688242)

9 (editorial or "case reports").pt. (667420)

10 (case stud$ or case report$).ti. (368542)

11 or/8-10 (5710891)

12 7 not 11 (69865)

13 limit 12 to yr="2020" (52783)

14 exp China/ or exp Chinese/ (292442)

15 13 and 14 (4232)

16 remove duplicates from 15 (4125)

**Database: CNKI**

**Search Strategy:**

| **#** | **Searches** | **Results** |
| --- | --- | --- |
| **1** | (主题=“新型冠状病毒” OR 主题=“COVID-19” OR 主题=“新冠肺炎 ”OR 主题=“新冠病毒”) AND 发表时间=2020-01-01 – 2020-12-31 | 13.47~13.49万 （12.07万） |
| **2** | 1 AND篇关摘=危重 OR 篇关摘=重 （模糊） | 2823 （2683） |
| **3** | 2 AND篇关摘=临床 OR 篇关摘=预后 （模糊） | 1204 |

**Database: Wangfang**

**Search Strategy:**

| **#** | **Searches** | **Results** |
| --- | --- | --- |
| **1** | (中英文扩展：(主题:("新型冠状病毒")+主题:("新冠病毒")+主题:("新冠肺炎")+主题:("COVID-19"))*(题名或关键词:(危重)+题名或关键词:(重))*(题名或关键词:(临床)+题名或关键词:(预后)）)*Date:2020- | 214 |

**Database: CMAPH**

**Search Strategy:**

| **#** | **Searches** | **Results** |
| --- | --- | --- |
| **1** | ((((((主题=“新型冠状病毒”) OR 主题=“新冠病毒”) OR 主题=“新冠肺炎”) OR 主题=“COVID-19” AND 发表时间=2020-2020) AND ((摘要=重) OR 摘要=危重 AND 发表时间=2020-2020)) AND ((摘要=临床) OR （摘要=预后 AND 发表时间=2020-2020） | 35 |
