# Supplementary material for: A look back at the first wave of COVID-19 in China: A systematic review and meta-analysis of mortality and health care resource use among severe or critical patients
Source: PLoS One. 2022 Mar 11;17(3):e0265117. doi: 10.1371/journal.pone.0265117 (PMC8916647; doi:10.1371/journal.pone.0265117)
Supplement: S2 Appendix — (DOCX) [file pone.0265117.s002.docx]

**S2 Appendix. Number of studies and characteristics of patients included in the meta-analysis**

| **Outcomes** | **Group of patients** | **Number of included studies** | **Number of events** | **Number of patients** | **Severe, n(%)** | **Male, n(%)** | **Age, mean ± SD** |
| --- | --- | --- | --- | --- | --- | --- | --- |
| **Primary outcomes** | | | | | | | |
| CFR, % | Whole group | 20 | 1201 | 7136 | 5765 (82.6) | 3248 (53.7) | 61.4 ± 14 |
|  | Severe subgroup | 9 | 629 | 3969 | 3969 (100) | 1629 (51.7) | 60.8 ± 14.3 |
|  | Critical subgroup | 4 | 578 | 1235 | 0 (0) | 790 (64.0) | 64.8 ± 14.2 |
|  | Whole group, December enrolment | 5 | 546 | 2531 | 1908 (75.4) | 1338 (52.9) | 60.3 ± 15.1 |
|  | Whole group, January enrolment | 11 | 643 | 4361 | 3686 (86.6) | 1764 (53.9) | 62.6 ± 12.8 |
|  | Whole group, February enrolment | 4 | 12 | 244 | 171 (89.5) | 146 (59.8) | 61.7 ± 13.3 |
|  | Hubei | 15 | 1175 | 6719 | 5421 (82) | 3002 (53.3) | 61.8 ± 13.7 |
|  | Outside Hubei | 4 | 12 | 244 | 171 (89.5) | 146 (59.8) | 58.4 ± 13.4 |
| Discharge rate, % | Whole group | 12 | 2145 | 3645 | 2881 (79) | 2015 (55.3) | 62 ± 13.5 |
|  | Severe subgroup | 4 | 237 | 1219 | 1219 (100) | 678 (55.6) | 62.7 ± 13.6 |
|  | Critical subgroup | 5 | 111 | 453 | 0 (0) | 305 (67.3) | 64.8 ± 13.6 |
| Use of invasive ventilation, % | Whole group | 17 | 548 | 4108 | 3032 (74.8) | 2269 (55.2) | 62.3 ± 13.8 |
|  | Severe subgroup | 8 | 196 | 1653 | 1653 (100) | 929 (55.9) | 61.1 ± 14.2 |
|  | Critical subgroup | 3 | 525 | 1154 | 0 (0) | 746 (64.6) | 63.5 ± 13.0 |
| Length of stay, days | Whole group | 19 | NA | 6807 | 5133 (75.4) | 3722 (54.7) | 61.7 ± 13.9 |
|  | Whole group, early phase of pandemic | 4 | NA | 2952 | 1908 (64.6) | 1627 (55.1) | 61.4 ± 14.4 |
|  | Whole group mid phase of pandemic | 11 | NA | 2047 | 1582 (79.3) | 1161 (56.7) | 62.5 ± 13.5 |
|  | Whole group, late phase of pandemic | 4 | NA | 1808 | 1643 (90.9) | 934 (51.7) | 61.2 ± 13.5 |
|  | Severe subgroup | 10 | NA | 3518 | 3518 (100) | 1843 (52.2) | 61.6 ± 13.9 |
|  | Critical subgroup | 4 | NA | 1250 | 0 (0) | 799 (64.0) | 64.5 ± 14.2 |
| **Secondary outcomes** | | | | | | | |
| Use of non-invasive ventilation, % | Whole group | 15 | 1029 | 3894 | 2805 (72) | 2191 (56.3) | 62.3 ± 13.8 |
| Use of ECMO, % | Whole group | 12 | 77 | 2390 | 1308 (54.7) | 1432 (60) | 61.5 ± 14.5 |

Abbreviations: CFR: case fatality ratio; ECMO: extracorporeal membrane oxygenation.
