# Supplementary material for: A look back at the first wave of COVID-19 in China: A systematic review and meta-analysis of mortality and health care resource use among severe or critical patients
Source: PLoS One. 2022 Mar 11;17(3):e0265117. doi: 10.1371/journal.pone.0265117 (PMC8916647; doi:10.1371/journal.pone.0265117)
Supplement: S3 Appendix — (DOCX) [file pone.0265117.s003.docx]

**S3 Appendix. Comorbidities of patients with severe or critical COVID-19 in each study included in the meta-analysis, n= 42**

| **Study name** | **Number of patients** | **Comorbidities, n(%)** | | | | | | | | |
| --- | --- | --- | --- | --- | --- | --- | --- | --- | --- | --- |
|  |  | Hypertension | Diabetes | CEVD | CVD | CPD | CKD | CLD | Cancer | With at least one comorbidity |
| *Guan et al. 2020 ( #5611 )^18^ | 173 | 41 ( 23.7 ) | 28 ( 16.2 ) | 4 ( 2.3 ) | 10 ( 5.8 ) | 6 ( 3.5 ) | 3 ( 1.7 ) | 1 ( 0.6 ) | 3 ( 1.7 ) | 67 ( 38.7 ) |
| Tang et al. 2020 ( #10371 )^34^ | 73 | 38 ( 52.1 ) | 20 ( 27.4 ) | NR | 23 ( 31.5 ) | 1 ( 1.4 ) | 3 ( 4.1 ) | NR | NR | NR |
| Xu et al. 2020 ( #9329 )^35^ | 107 | 35 ( 32.7 ) | NR | NR | NR | 1 ( 0.9 ) | 6 ( 5.6 ) | NR | NR | NR |
| Liu et al. 2020 ( #3961 )^36^ | 349 | 119 ( 34.1 ) | 62 ( 17.8 ) | 21 ( 6 ) | 37 ( 10.6 ) | 13 ( 3.7 ) | 10 ( 2.9 ) | 12 ( 3.4 ) | 17 ( 4.9 ) | NR |
| *Wu et al. 2020 ( #2644 )^37^ | 1763 | 461 ( 26.1 ) | 237 ( 13.4 ) | NR | NR | 66 ( 3.7 ) | 44 ( 2.5 ) | NR | 28 ( 1.6 ) | NR |
| *Xu et al. 2020 ( #4838 )^38^ | 239 | 105 ( 43.9 ) | 44 ( 18.4 ) | 13 ( 5.4 ) | 35 ( 14.6 ) | 12 ( 5 ) | NR | 20 ( 8.4 ) | 13 ( 5.4 ) | NR |
| Zhang et al. 2020 ( #10522 )^39^ | 107 | 37 ( 34.6 ) | 13 ( 12.1 ) | 9 ( 8.4 ) | 16 ( 15 ) | 6 ( 5.6 ) | 6 ( 5.6 ) | 6 ( 5.6 ) | 6 ( 5.6 ) | 68 ( 63.6 ) |
| Liu et al. 2020 ( #3799 )^40^ | 79 | 31 ( 39.2 ) | 13 ( 16.5 ) | NR | 6 ( 7.6 ) | 5 ( 6.3 ) | 2 ( 2.5 ) | NR | NR | NR |
| Cai et al. 2020 ( #4791 )^14^ | 58 | 22 ( 37.9 ) | 8 ( 13.8 ) | NR | 13 ( 22.4 ) | NR | NR | 8 ( 13.8 ) | 2 ( 3.4 ) | NR |
| *Ma et al. 2020 ( #2629 )^43^ | 82 | 31 ( 37.8 ) | 18 ( 22 ) | 5 ( 6.1 ) | 10 ( 12.2 ) | 6 ( 7.3 ) | 1 ( 1.2 ) | 3 ( 3.7 ) | NR | NR |
| *Zhang et al. 2020 ( #4274 )^64^ | 539 | NR | NR | NR | NR | NR | NR | NR | NR | NR |
| Wang et al. 2020 ( #10019 )^63^ | 239 | NR | NR | NR | NR | NR | NR | NR | NR | NR |
| Xu et al. 2020 ( #3248 )^47^ | 50 | 24 ( 48 ) | 12 ( 24 ) | NR | 4 ( 8 ) | 0 ( 0 ) | 3 ( 6 ) | NR | 3 ( 6 ) | NR |
| Liu et al. 2020 ( #2726 )^42^ | 957 | 453 ( 47.3 ) | 201 ( 21 ) | NR | 120 ( 12.5 ) | 15 ( 1.6 ) | 18 ( 1.9 ) | 34 ( 3.6 ) | 38 ( 4 ) | 635 ( 66.4 ) |
| *Chen et al. 2020 ( #4319 )^53^ | 51 | 27 ( 52.9 ) | 11 ( 21.6 ) | 5 ( 9.8 ) | 12 ( 23.5 ) | 8 ( 15.7 ) | 2 ( 3.9 ) | 3 ( 5.9 ) | 1 ( 2 ) | NR |
| Yu et al. 2020 ( #10679 )^65^ | 53 | 21 ( 39.6 ) | 18 ( 34 ) | 2 ( 3.8 ) | 10 ( 18.9 ) | 2 ( 3.8 ) | NR | 6 ( 11.3 ) | 2 ( 3.8 ) | 41 ( 77.4 ) |
| *Wang et al. 2020 ( #3550 )^32^ | 236 | 102 ( 43.2 ) | 56 ( 23.7 ) | NR | 17 ( 7.2 ) | NR | NR | NR | NR | NR |
| *Zhu et al. 2020 ( #8898 )^16^ | 102 | NR | NR | NR | NR | NR | NR | NR | NR | NR |
| Xia et al. 2020 ( #8941 )^62^ | 1568 | 561 ( 35.8 ) | 249 ( 15.9 ) | 87 ( 5.5 ) | 237 ( 15.1 ) | 103 ( 6.6 ) | 37 ( 2.4 ) | 43 ( 2.7 ) | 57 ( 3.6 ) | NR |
| Liu et al. 2020 ( #4147 )^50^ | 311 | NR | NR | NR | NR | NR | NR | NR | NR | NR |
| Zhang et al. 2020 ( #3150 )^48^ | 75 | 36 ( 48 ) | 15 ( 20 ) | 8 ( 10.7 ) | 13 ( 17.3 ) | 3 ( 4 ) | NR | 1 ( 1.3 ) | 5 ( 6.7 ) | NR |
| Pan et al. 2020 ( #8173 )^59^ | 124 | 62 ( 50 ) | 25 ( 20.2 ) | NR | 19 ( 15.3 ) | 11 ( 8.9 ) | NR | NR | NR | NR |
| Yu et al. 2020 ( #2842 )^44^ | 864 | 205 ( 23.7 ) | 160 ( 18.5 ) | 33 ( 3.8 ) | 85 ( 9.8 ) | 37 ( 4.3 ) | 15 ( 1.7 ) | 22 ( 2.5 ) | 13 ( 1.5 ) | NR |
| Xiong et al. 2020 ( #11162 )^66^ | 305 | 153 ( 50.2 ) | 76 ( 24.9 ) | 40 ( 13.1 ) | 51 ( 16.7 ) | 28 ( 9.2 ) | 32 ( 10.5 ) | NR | 10 ( 3.3 ) | NR |
| Yang et al. 2020 ( #5850 )^57^ | 52 | NR | 9 ( 17.3 ) | 7 ( 13.5 ) | 5 ( 9.6 ) | 4 ( 7.7 ) | NR | NR | 2 ( 3.8 ) | NR |
| *Zhou et al. 2020 ( #4245 )^55^ | 195 | NR | NR | NR | NR | NR | NR | NR | NR | 137 ( 70.3 ) |
| Cheng et al. 2020 ( #11809 )^67^ | 181 | 53 ( 29.3 ) | 20 ( 11 ) | 8 ( 4.4 ) | 16 ( 8.8 ) | 12 ( 6.6 ) | 6 ( 3.3 ) | 5 ( 2.8 ) | 6 ( 3.3 ) | NR |
| Shao et al. 2020 ( #6230 )^17^ | 136 | 41 ( 30.1 ) | 27 ( 19.9 ) | 5 ( 3.7 ) | 15 ( 11 ) | 6 ( 4.4 ) | 3 ( 2.2 ) | NR | 10 ( 7.4 ) | NR |
| *Xie et al. 2020 ( #4193 )^52^ | 733 | 308 ( 42 ) | 138 ( 18.8 ) | 34 ( 4.6 ) | 108 ( 14.7 ) | 37 ( 5 ) | 13 ( 1.8 ) | 11 ( 1.5 ) | 24 ( 3.3 ) | 454 ( 62 ) |
| *Ma et al. 2020 ( #8986 )^60^ | 72 | 15 ( 20.8 ) | 16 ( 22.2 ) | NR | 5 ( 6.9 ) | 4 ( 5.6 ) | 0 ( 0 ) | 5 ( 6.9 ) | 0 ( 0 ) | NR |
| Wang et al. 2020 ( #8872 )^61^ | 59 | 8 ( 13.6 ) | 3 ( 5.1 ) | 2 ( 3.4 ) | NR | 1 ( 1.7 ) | NR | NR | 0 ( 0 ) | NR |
| *Huang et al. 2020 ( #2956 )^45^ | 60 | 14 ( 23.3 ) | 10 ( 16.7 ) | 4 ( 6.7 ) | 3 ( 5 ) | 2 ( 3.3 ) | 1 ( 1.7 ) | NR | NR | 31 ( 51.7 ) |
| Zhong et al. 2020 ( #10923 )^68^ | 583 | NR | 69 ( 11.8 ) | NR | 211 ( 36.2 ) | 28 ( 4.8 ) | NR | 9 ( 1.5 ) | NR | NR |
| Yang et al. 2020 ( #10707 )^69^ | 301 | 105 ( 34.9 ) | 43 ( 14.3 ) | NR | NR | NR | NR | NR | NR | 173 ( 57.5 ) |
| Chen et al. 2020 ( #3846 )^15^ | 681 | 293 ( 43 ) | 114 ( 16.7 ) | 33 ( 4.8 ) | 80 ( 11.7 ) | 15 ( 2.2 ) | 27 ( 4 ) | NR | NR | NR |
| *Xu et al. 2020 ( #3634 )^49^ | 198 | 107 ( 54 ) | 35 ( 17.7 ) | 6 ( 3 ) | 13 ( 6.6 ) | 4 ( 2 ) | NR | NR | 10 ( 5.1 ) | 134 ( 67.7 ) |
| *Zhang et al. 2020 ( #4326 )^54^ | 78 | 34 ( 43.6 ) | 12 ( 15.4 ) | NR | 2 ( 2.6 ) | 3 ( 3.8 ) | 2 ( 2.6 ) | 4 ( 5.1 ) | 3 ( 3.8 ) | NR |
| Zhang et al. 2020 ( #4544 )^56^ | 136 | 68 ( 50 ) | 56 ( 41.2 ) | NR | 26 ( 19.1 ) | 12 ( 8.8 ) | 5 ( 3.7 ) | 1 ( 0.7 ) | 8 ( 5.9 ) | NR |
| *Tian et al. 2020 ( #3869 )^51^ | 148 | 68 ( 45.9 ) | 33 ( 22.3 ) | NR | 23 ( 15.5 ) | 2 ( 1.4 ) | 4 ( 2.7 ) | 4 ( 2.7 ) | 148 ( 100 ) | NR |
| Li et al. 2020 ( #3139 )^46^ | 173 | 173 ( 100 ) | 76 ( 43.9 ) | 50 ( 28.9 ) | 47 ( 27.2 ) | NR | 30 ( 17.3 ) | NR | 8 ( 4.6 ) | NR |
| *Li et al. 2020 ( #2564 )^41^ | 103 | 56 ( 54.4 ) | 21 ( 20.4 ) | 18 ( 17.5 ) | 26 ( 25.2 ) | NR | 6 ( 5.8 ) | 10 ( 9.7 ) | 3 ( 2.9 ) | NR |
| *Yu et al. 2020 ( #6374 )^58^ | 226 | 96 ( 42.5 ) | 47 ( 20.8 ) | 15 ( 6.6 ) | 32 ( 14.2 ) | 15 ( 6.6 ) | 8 ( 3.5 ) | 3 ( 1.3 ) | 11 ( 4.9 ) | 155 ( 68.6 ) |

Footnote: * indicates the study involves patients from multiple centers; otherwise, the study involves a single center.

Abbreviations: CEVD: cerebrovascular disease; CVD: cardiovascular disease; CPD: chronic pulmonary disease; CKD: chronic kidney disease; CLD: chronic liver disease; NR: not reported.
