# Supplementary material for: A look back at the first wave of COVID-19 in China: A systematic review and meta-analysis of mortality and health care resource use among severe or critical patients
Source: PLoS One. 2022 Mar 11;17(3):e0265117. doi: 10.1371/journal.pone.0265117 (PMC8916647; doi:10.1371/journal.pone.0265117)
Supplement: S4 Appendix — Table A. Risk of bias of included cohort studies. Table B. Risk of bias of included case-control studies. Table C. Risk of bias of included randomized controlled trials. (DOCX) [file pone.0265117.s004.docx]

**S4 Appendix. Quality assessment of included studies**

**Table A. Risk of bias of included cohort studies, n= 35**

| **Study name** | **From the same population** | **Assessment of exposure** | **Outcome nor present at start** | **Adjustment** | **Assessment of prognostic factors** | **Assessment of outcome** | **Adequate follow-up** | **Co-intervention similarity** |
| --- | --- | --- | --- | --- | --- | --- | --- | --- |
| *Guan et al. 2020 ( #5611 )^18^ | Definitely yes | Definitely yes | Probably yes | Probably no | Definitely yes | Definitely yes | Definitely yes | Probably no |
| Xu et al. 2020 ( #9329 )^35^ | Definitely yes | Definitely yes | Definitely yes | Definitely no | Definitely yes | Definitely yes | Probably yes | Probably no |
| Liu et al. 2020 ( #3961 )^36^ | Definitely yes | Definitely yes | Definitely yes | Probably no | Probably yes | Definitely yes | Probably yes | Probably no |
| *Wu et al. 2020 ( #2644 )^37^ | Definitely yes | Definitely yes | Probably yes | Definitely yes | Definitely yes | Definitely yes | Definitely yes | Probably no |
| *Xu et al. 2020 ( #4838 )^38^ | Definitely yes | Definitely yes | Definitely yes | Probably no | Definitely yes | Definitely yes | Definitely yes | Probably yes |
| Zhang et al. 2020 ( #10522 )^39^ | Definitely yes | Definitely yes | Definitely yes | Probably no | Definitely yes | Definitely yes | Definitely yes | Probably yes |
| Liu et al. 2020 ( #3799 )^40^ | Definitely yes | Definitely yes | Definitely yes | Probably yes | Definitely yes | Definitely yes | Probably yes | Probably yes |
| Cai et al. 2020 ( #4791 )^14^ | Definitely yes | Definitely yes | Definitely yes | Definitely yes | Definitely yes | Definitely yes | Definitely yes | Probably no |
| *Ma et al. 2020 ( #2629 )^43^ | Definitely yes | Definitely yes | Definitely yes | Probably yes | Definitely yes | Definitely yes | Probably yes | Probably no |
| *Zhang et al. 2020 ( #4274 )^64^ | Definitely yes | Definitely yes | Definitely yes | Probably no | Probably yes | Definitely yes | Probably yes | Probably no |
| Wang et al. 2020 ( #10019 )^63^ | Definitely yes | Definitely yes | Definitely yes | Probably no | Definitely yes | Definitely yes | Probably yes | Probably no |
| Xu et al. 2020 ( #3248 )^47^ | Probably yes | Definitely yes | Probably yes | Definitely yes | Definitely yes | Definitely yes | Probably yes | Definitely no |
| Liu et al. 2020 ( #2726 )^42^ | Definitely yes | Definitely yes | Definitely yes | Definitely yes | Definitely yes | Definitely yes | Definitely yes | Probably no |
| *Chen et al. 2020 ( #4319 )^53^ | Definitely yes | Definitely yes | Definitely yes | Probably no | Probably yes | Definitely yes | Definitely yes | Probably yes |
| Yu et al. 2020 ( #10679 )^65^ | Definitely yes | Definitely yes | Probably yes | Probably no | Definitely yes | Definitely yes | Definitely yes | Probably no |
| *Zhu et al. 2020 ( #8898 )^16^ | Definitely yes | Definitely yes | Probably yes | Probably yes | Definitely yes | Definitely yes | Definitely yes | Probably yes |
| Xia et al. 2020 ( #8941 )^62^ | Definitely yes | Definitely yes | Probably yes | Probably no | Definitely yes | Definitely yes | Definitely yes | Probably yes |
| Liu et al. 2020 ( #4147 )^50^ | Definitely yes | Definitely yes | Probably yes | Definitely yes | Definitely yes | Definitely yes | Definitely yes | Definitely yes |
| Zhang et al. 2020 ( #3150 )^48^ | Definitely yes | Definitely yes | Definitely yes | Probably yes | Probably yes | Definitely yes | Probably yes | Probably yes |
| Yu et al. 2020 ( #2842 )^44^ | Definitely yes | Definitely yes | Definitely yes | Probably no | Definitely yes | Definitely yes | Definitely yes | Probably no |
| Xiong et al. 2020 ( #11162 )^66^ | Definitely yes | Definitely yes | Probably yes | Probably no | Definitely yes | Definitely yes | Definitely yes | Probably yes |
| Yang et al. 2020 ( #5850 )^57^ | Definitely yes | Definitely yes | Definitely yes | Probably no | Definitely yes | Definitely yes | Definitely yes | Probably no |
| Cheng et al. 2020 ( #11809 )^67^ | Definitely yes | Definitely yes | Definitely yes | Probably no | Definitely yes | Definitely yes | Definitely yes | Definitely no |
| Shao et al. 2020 ( #6230 )^17^ | Definitely yes | Definitely yes | Definitely yes | Probably yes | Probably yes | Definitely yes | Probably yes | Probably yes |
| *Xie et al. 2020 ( #4193 )^52^ | Definitely yes | Definitely yes | Probably yes | Definitely yes | Definitely yes | Definitely yes | Definitely yes | Probably yes |
| *Ma et al. 2020 ( #8986 )^60^ | Definitely yes | Definitely yes | Definitely yes | Definitely no | Probably yes | Definitely yes | Probably yes | Definitely yes |
| Wang et al. 2020 ( #8872 )^61^ | Definitely yes | Definitely yes | Definitely yes | Definitely no | Definitely yes | Definitely yes | Probably yes | Probably yes |
| *Huang et al. 2020 ( #2956 )^45^ | Definitely yes | Definitely yes | Definitely yes | Definitely yes | Definitely yes | Definitely yes | Definitely yes | Definitely yes |
| Zhong et al. 2020 ( #10923 )^68^ | Definitely yes | Definitely yes | Probably yes | Probably no | Definitely yes | Definitely yes | Definitely yes | Definitely yes |
| Yang et al. 2020 ( #10707 )^69^ | Definitely yes | Definitely yes | Probably yes | Probably no | Definitely yes | Definitely yes | Definitely yes | Probably no |
| Chen et al. 2020 ( #3846 )^15^ | Definitely yes | Definitely yes | Definitely yes | Definitely yes | Definitely yes | Definitely yes | Probably yes | Probably yes |
| *Xu et al. 2020 ( #3634 )^49^ | Definitely yes | Definitely yes | Probably yes | Probably no | Definitely yes | Definitely yes | Definitely yes | Probably no |
| *Zhang et al. 2020 ( #4326 )^54^ | Definitely yes | Definitely yes | Definitely yes | Definitely no | Definitely yes | Probably yes | Probably yes | Probably no |
| Zhang et al. 2020 ( #4544 )^56^ | Definitely yes | Definitely yes | Definitely yes | Definitely yes | Definitely yes | Definitely yes | Definitely yes | Probably yes |
| *Tian et al. 2020 ( #3869 )^51^ | Definitely yes | Definitely yes | Probably yes | Definitely yes | Definitely yes | Definitely yes | Definitely yes | Probably no |

Footnote: * indicates the study involves patients from multiple centers; otherwise, the study involves a single center.

**Table B. Risk of bias of included case-control studies, n=2**

| **Study name** | **Assessment of exposure** | **Assessment of outcome** | **Assessment of cases** | **Assessment of controls** | **Matching between cases and controls** |
| --- | --- | --- | --- | --- | --- |
| Pan et al. 2020 ( #8173 )^59^ | Definitely yes | Definitely yes | Definitely yes | Definitely yes | Probably yes |
| Tang et al. 2020 ( #10371 )^34^ | Definitely yes | Definitely yes | Definitely yes | Definitely yes | Definitely yes |

**Table C. Risk of bias of included randomized controlled trials, n=2**

| **Study name** | **Sequence Generation** | **Allocation Sequence Concealment** | **Blinding** | | | | | **Missing Outcome Data** | **Free of selective outcome**  **reporting** |
| --- | --- | --- | --- | --- | --- | --- | --- | --- | --- |
|  |  |  | **Patients** | **Healthcare providers** | **Data collectors** | **Outcome assessors** | **data analysts** |  |  |
| *Wang et al. 2020 ( #3550 )^32^ | Definitely yes | Definitely yes | Definitely yes | Definitely yes | Probably yes | Probably yes | Probably yes | Definitely yes | Definitely yes |
| *Li et al. 2020 ( #2564 )^41^ | Definitely yes | Probably no | Definitely no | Definitely no | Probably no | Definitely yes | Probably no | Definitely yes | Definitely yes |

Footnote: * indicates the study involves patients from multiple centers.
