# Supplementary material for: A look back at the first wave of COVID-19 in China: A systematic review and meta-analysis of mortality and health care resource use among severe or critical patients
Source: PLoS One. 2022 Mar 11;17(3):e0265117. doi: 10.1371/journal.pone.0265117 (PMC8916647; doi:10.1371/journal.pone.0265117)
Supplement: S5 Appendix — (DOCX) [file pone.0265117.s005.docx]

**S5 Appendix. Heterogeneity and publication bias assessment for meta-analyses for primary outcomes**

| **Meta-analysis** | **Number of studies** | **Heterogeneity** | **Egger’s test** |
| --- | --- | --- | --- |
| CFR in total population | 20 | 𝜏^2^ = 0.02 [0.01; 0.05],  Q = 892.30, df = 19, p < 0.0001,  I^2^ = 97.9% [97.4%; 98.3%]. | t = 3.21, df = 18, p-value = 0.0048 |
| Length of stay in total population | 19 | 𝜏^2^ = 29.07 [16.33; 64.25],  Q = 1352.67, df = 18, p < 0.0001,  I^2^ = 98.7% [98.4%; 98.9%] | t = 2.09, df = 17, p-value = 0.0519 |
| Discharge rate in total population | 12 | 𝜏^2^ = 0.10 [0.05; 0.28],  Q = 6397.15, df = 11, p < 0.0001, I^2^ = 99.8% | t = -1.78, df = 10, p-value = 0.1059 |
| Intensive ventilation rate in total population | 17 | 𝜏^2^ = 0.02 [0.01; 0.05],  Q = 484.68, df = 16, p < 0.0001,  I^2^ = 96.7% [95.7%; 97.5%] | t = 4.58, df = 15, p-value = 0.0004 |
