# Supplementary material for: A look back at the first wave of COVID-19 in China: A systematic review and meta-analysis of mortality and health care resource use among severe or critical patients
Source: PLoS One. 2022 Mar 11;17(3):e0265117. doi: 10.1371/journal.pone.0265117 (PMC8916647; doi:10.1371/journal.pone.0265117)
Supplement: S6 Appendix — Fig A 28-day and 14-day CFRs among patients with severe vs critical COVID-19. Fig B 28-day and 14-day CFRs in Hubei vs other locations in China. Fig C Length of hospital stay among patients with severe vs critical COVID-19. Fig D Discharge rate among patients with severe vs critical COVID-19. Fig E The use of invasive ventilation rate among patients with severe vs critical COVID-19. (DOCX) [file pone.0265117.s006.docx]

**S6 Appendix. Subgroup analysis results**


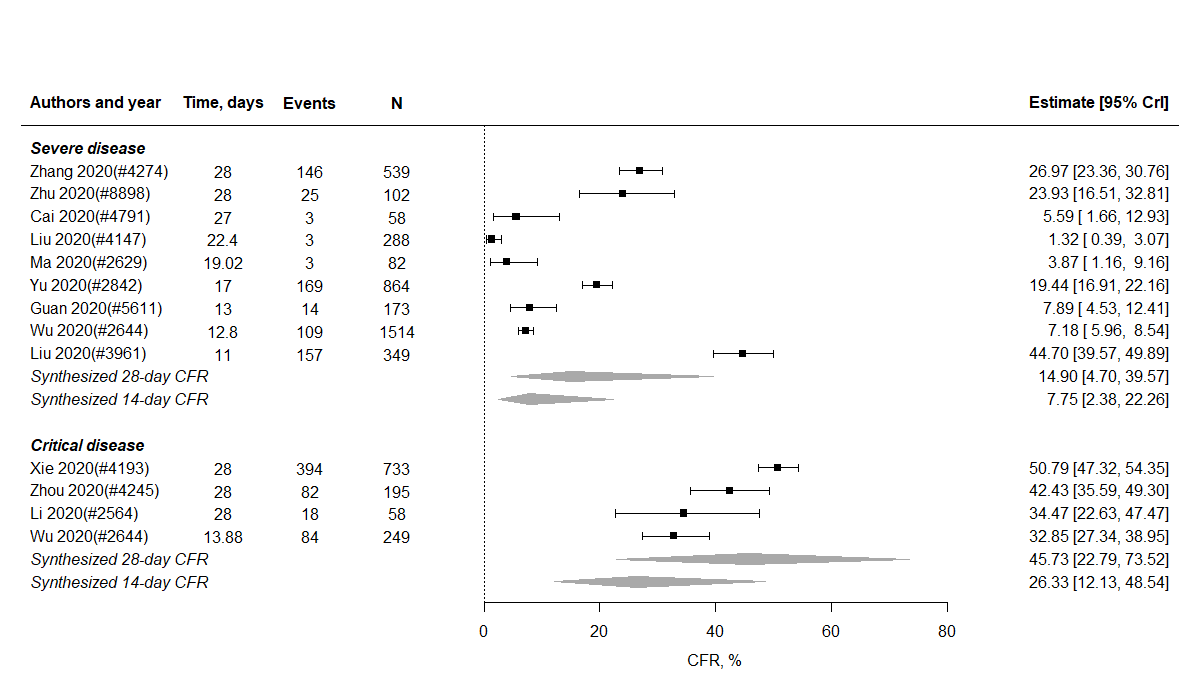


**Fig. A 28-day and 14-day CFRs among patients with severe vs critical COVID-19 in China**


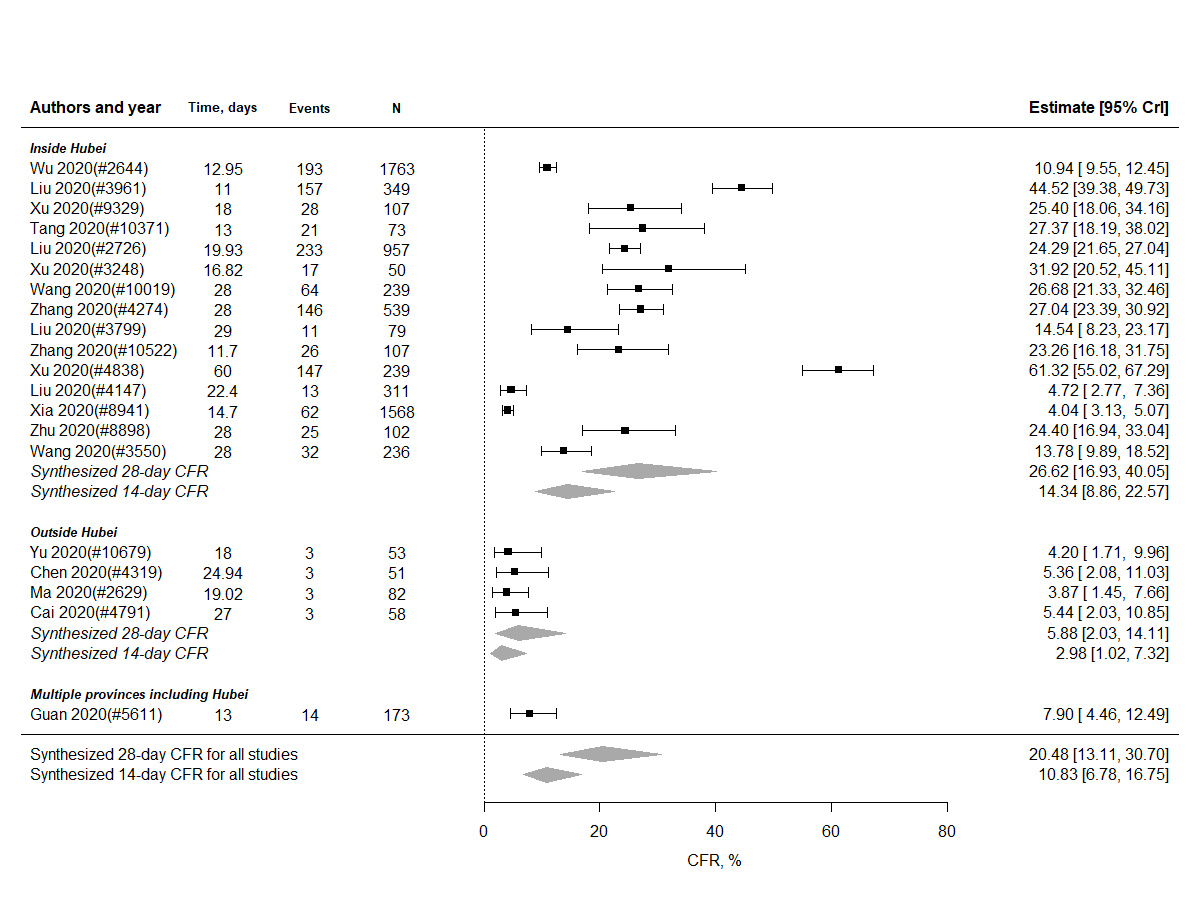


**Fig. B 28-day and 14-day CFRs in Hubei vs other locations in China**

**
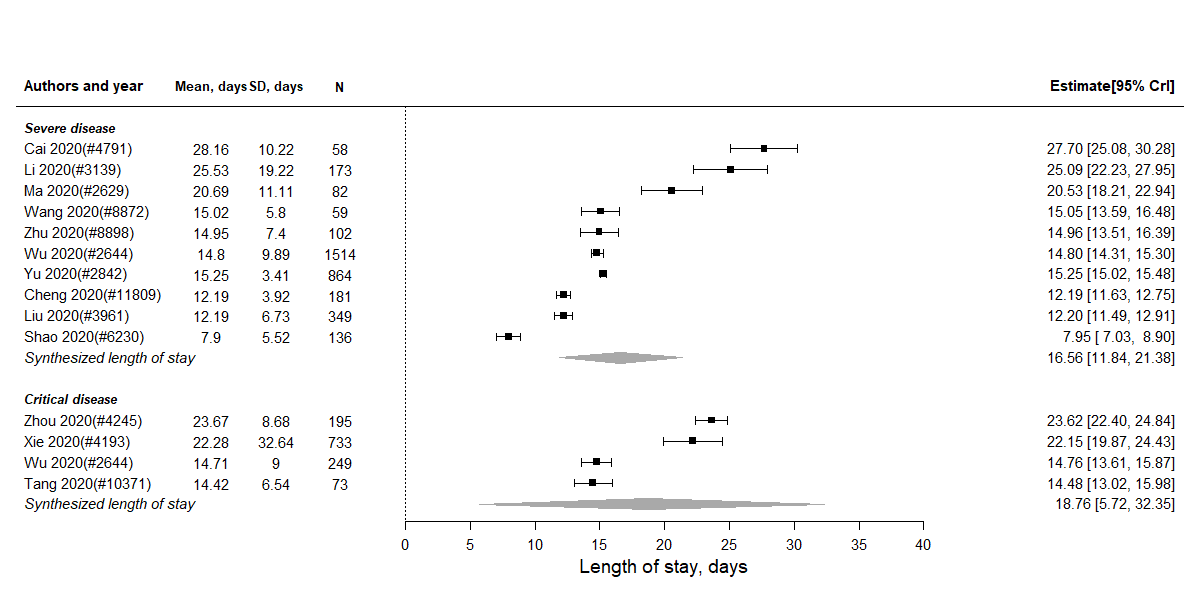
**

**Fig. C Length of hospital stay among patients with severe vs critical COVID-19 in China**

**
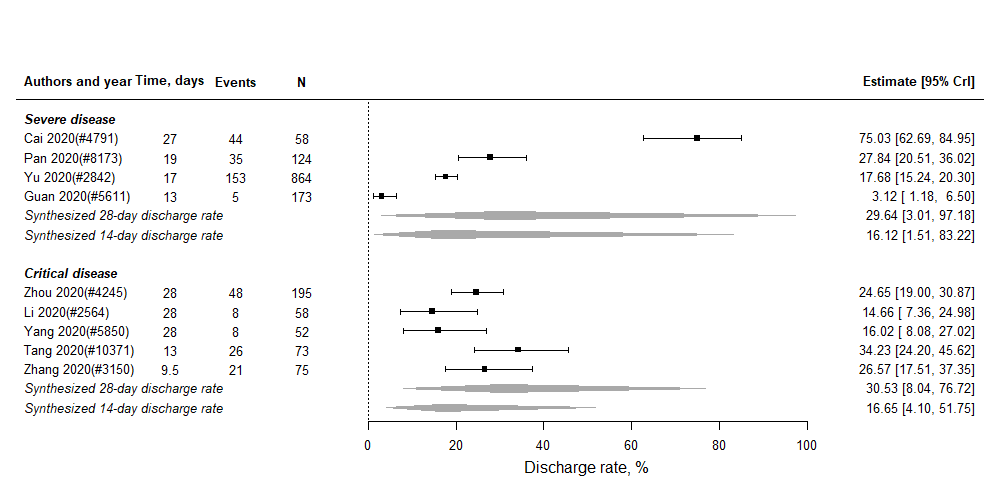
**

**Fig. D Discharge rate among patients with severe vs critical COVID-19 in China**

**
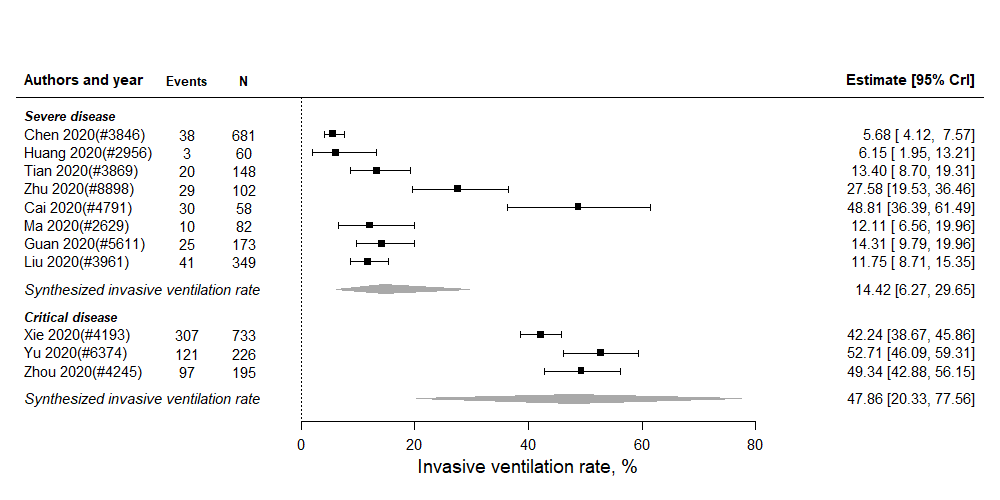
**

**Fig. E The use of invasive ventilation rate among patients with severe vs critical COVID-19 in China**
