# Supplementary material for: A look back at the first wave of COVID-19 in China: A systematic review and meta-analysis of mortality and health care resource use among severe or critical patients
Source: PLoS One. 2022 Mar 11;17(3):e0265117. doi: 10.1371/journal.pone.0265117 (PMC8916647; doi:10.1371/journal.pone.0265117)
Supplement: S7 Appendix — (DOCX) [file pone.0265117.s007.docx]

**S7 Appendix. Sensitivity analyses with different priors for meta-analyses for primary outcomes**

| **Meta-analysis** | **Priors** | **Pooled estimates** |
| --- | --- | --- |
| CFR in total population | sd.m ~ dunif (1/1000, 10) | 28-day CFR: 20.45% (95% CrI: 13.06, 30.62)  14-day CFR: 10.81% (95% CrI: 6.76, 16.71) |
|  | sd.m ~ dunif (1/1000, 2) | 28-day CFR: 20.44% (95% CrI: 13.01, 30.54)  14-day CFR: 10.81% (95% CrI: 6.73, 16.66) |
|  | tau.m ~ dgamma (0.001, 0.001) | 28-day CFR: 20.52% (95% CrI:13.22, 30.29)  14-day CFR: 10.85% (95% CrI: 6.84, 16.50) |
|  | tau.m ~ dgamma (0.0001, 0.0001) | 28-day CFR: 20.50% (95% CrI: 13.3, 30.16)  14-day CFR: 10.83% (95% CrI: 6.90, 16.43) |
| Length of stay in total population | sd.m ~ dunif (1/1000, 10) | 18.46 (95% CrI: 15.65, 21.21) |
|  | sd.m ~ dunif (1/1000, 2) | 18.15 (95% CrI: 17.20, 19.09) |
|  | tau.m ~ dgamma (0.001, 0.001) | 18.46 (95% CrI: 15.79, 21.18) |
|  | tau.m ~ dgamma (0.0001, 0.0001) | 18.45 (95% CrI: 15.80, 21.15) |
| Discharge rate in total population | sd.m ~ dunif (1/1000, 10) | 28-day discharge rate: 50.47% (95% CrI: 25.74, 79.84)  14-day discharge rate: 29.62% (95% CrI: 13.82, 55.10) |
|  | sd.m ~ dunif (1/1000, 2) | 28-day discharge rate: 50.47% (95% CrI: 26.93, 78.27)  14-day discharge rate: 29.63% (95% CrI: 14.52, 53.38) |
|  | tau.m ~ dgamma (0.001, 0.001) | 28-day discharge rate: 50.64% (95% CrI: 27.41, 78.33)  14-day discharge rate: 29.74% (95% CrI: 14.80, 53.45) |
|  | tau.m ~ dgamma (0.0001, 0.0001) | 28-day discharge rate: 50.64% (95% CrI: 27.82, 78.13)  14-day discharge rate: 29.74% (95% CrI: 15.04, 53.24) |
| Intensive ventilation rate in total population | sd.m ~ dunif (1/1000, 10) | 13.45% (95% CrI: 7.62, 22.31) |
|  | sd.m ~ dunif (1/1000, 2) | 13.44% (95% CrI: 7.66, 22.41) |
|  | tau.m ~ dgamma (0.001, 0.001) | 13.47% (95% CrI: 7.82, 22.17) |
|  | tau.m ~ dgamma (0.0001, 0.0001) | 13.44% (95% CrI: 7.74, 22.12) |

Abbreviations: CrI: credential intervals
